# Case Report: Identification of Novel Variants in *ERCC4* and *DDB2* Genes in Two Tunisian Patients With Atypical Xeroderma Pigmentosum Phenotype

**DOI:** 10.3389/fgene.2021.650639

**Published:** 2021-05-31

**Authors:** Imen Nabouli, Asma Chikhaoui, Houcemeddine Othman, Sahar Elouej, Meriem Jones, Arnaud Lagarde, Meriem Ben Rekaya, Olfa Messaoud, Mohamed Zghal, Valerie Delague, Nicolas Levy, Annachiara De Sandre-Giovannoli, Sonia Abdelhak, Houda Yacoub-Youssef

**Affiliations:** ^1^Laboratoire de Génomique Biomédicale et Oncogénétique, Institut Pasteur de Tunis, LR16IPT05, Université Tunis ElManar, Tunis, Tunisia; ^2^Faculty of Health Sciences, Sydney Brenner Institute for Molecular Bioscience, University of the Witwatersrand, Johannesburg, South Africa; ^3^Aix Marseille Univ, INSERM, MMG, U1251, Marseille, France; ^4^Service de dermatologie, Hôpital Charles Nicolle, Tunis, Tunisia; ^5^Departement of Medical Genetics, Assistance Publique Hôpitaux de Marseille, La Timone Children's Hospital, Marseille, France; ^6^Biological Resource Center (CRB-TAC), Assistance Publique Hôpitaux de Marseille, La Timone Children's Hospital, Marseille, France

**Keywords:** xeroderma pigmentosum, NER defects, skin cancer, ERCC4/XPF, DDB2 gene

## Abstract

Xeroderma Pigmentosum (XP) is a rare genetic disorder affecting the nucleotide excision repair system (NER). It is characterized by an extreme sensitivity to sunlight that induces cutaneous disorders such as severe sunburn, freckling and cancers. In Tunisia, six complementation groups have been already identified. However, the genetic etiology remains unknown for several patients. In this study, we investigated clinical characteristics and genetic defects in two families with atypical phenotypes originating from the central region in Tunisia. Clinical investigation revealed mild cutaneous features in two patients who develop multiple skin cancers at later ages, with no neurological disorders. Targeted gene sequencing revealed that they carried novel variants. A homozygous variation in the *ERCC4* gene c.1762G>T, p.V588F, detected in patient XP21. As for patient XP134, he carried two homozygous mutations in the *DDB2* gene c.613T>C, p.C205R and c.618C>A, p.S206R. Structural modeling of the protein predicted the identified *ERCC4* variant to mildly affect protein stability without affecting its functional domains. As for the case of DDB2 double mutant, the second variation seems to cause a mild effect on the protein structure unlike the first variation which does not seem to have an effect on it. This study contributes to further characterize the mutation spectrum of XP in Tunisian families. Targeted gene sequencing accelerated the identification of rare unexpected genetic defects for diagnostic testing and genetic counseling.

## Introduction

Consanguinity plays a relevant role in the emergence of rare genetic diseases especially in North Africa and Middle East countries (Romdhane et al., 2012). Xeroderma pigmentosum (XP) is a rare autosomal recessive disorder characterized by predisposition to cutaneous malignancies. Its prevalence is estimated to 1/250.000 in Europe (Kleijer et al., 2008). However, it is more frequent in North Africa and especially in Tunisia (1/10.000) (Zghal et al., 2005). XP displays a wide clinical and genetic heterogeneity. It results from mutations in eight different genes: *XPA* through *XPG*, which encode Nucleotide Excision Repair (NER) genes, and *XPV* that encodes the Translesion Synthesis (TLS) DNA polymerase eta (Kleijer et al., 2008).

XP prognosis was widely improved due to increased knowledge about the frequent forms of the disease in Tunisia as XP-A, XP-C and XP-V (Ben Rekaya et al., 2009, 2014; Messaoud et al., 2010), Recently, thanks to the use of novel technologies such as high throughput sequencing, extremely rare forms are increasingly being identified as XP-D, XP-E, XP-G (Ben Rekaya et al., 2017; Chikhaoui et al., 2019).

The identification of disease-causing mutations in XP patients has an important impact on patient care, as it provides early dermatological follow up to prevent skin cancers and provides genetic counseling for affected family members. However, genetic diagnosis is difficult to carry out for a disease that shows such genetic heterogeneity. In addition, Sanger sequencing for known recurrent mutations that are common in specific geographic area does not always succeed in finding the genetic causes of atypical forms of the disease.

In this study, we describe clinical features and report novel genetic defects using targeted gene sequencing in two patients with particular forms of XP. One associated to XP-F, phenotype which we identify for the first time in the Tunisian population and the other consists of a double homozygous mutation in the *DDB2* gene associated to XP-E form.

### Case Presentation

#### Clinical Presentation

##### Patient XP21

**Figure 1:**
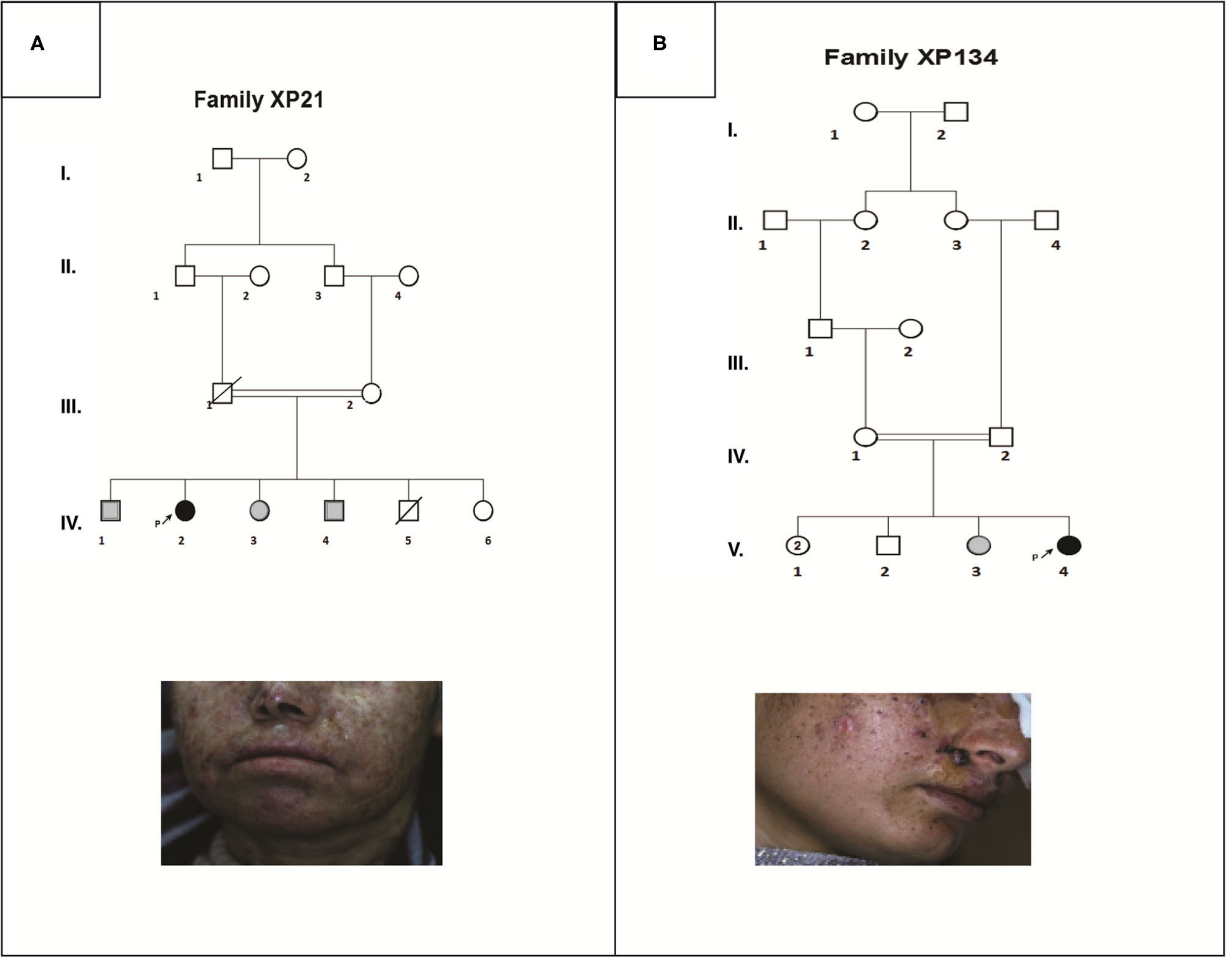
Pedigrees and clinical images describing two XP affected family members (XP21 and XP134) **(A,B)**. Filled symbols represent affected individuals, open symbols represent unaffected individuals, gray symbols are for suspected individuals, the studied pro-band is indicated with an arrow.

Patient XP21 is a 56-year-old woman, second child of healthy first-degree consanguineous parents originating from North-West of Tunisia (Figure 1A). When she was 6 years old, she developed achromic maculas on sun-exposed area of her skin. She consulted a doctor at 26, when she developed her fist basal cell carcinoma (BCC). XP21 had a normal psychomotor and mental development with no neurological manifestations. In her last consultation at age 56, when this study was conducted, she had developed several actinic keratosis. Her skin was dry and presented hyperpigmented areas. It is important to note that XP21 did not suffer from acute sunburn reaction nor had any photosensitivity manifestations. In addition, she had a short stature, but no microcephaly or neurological disorders suggestive of Cockayne syndrome were observed (Table 1). Only the reported patient and her healthy sister underwent clinical examination in a medical center. As mentioned by the patient, other family members with similar phenotypes came to our attention during the genetic inquiry about familial history, who presented mainly achromic maculas on their sun exposed area and that's in 2 brothers and one sister (Figure 1A). Unfortunately, they were out of reach as they lived far from medical centers.

**Table 1:** Summary of the clinical examination of XP patients.

	**XP21 (XP-F)**	**XP134-1 (XP-E)**
Age at last observation (year)	56	15
Age at onset of first symptoms (year)	6	5
Sex	F	F
Protection level	+	–
Geographical origin	Seliana (North West)	Kairouan (Sbikha)
Photophobia	–	±
Photosensitivity	–	–
Neurologic phenotype	–	–
Language delay	–	–
Sunburn	–	–
Irritation	±	±
Hyperpigmentation	+	+
Hypopigmented macula	–	+
Achromic macula	+	+
Dry skin	+	+
Cheilit	+	±
Age at consultation for skin cancer	26 years old for BCC	15 years old Multiple actinic keratosis, SCC and BCC
Actinic keratosis	+	++
Benign skin neoplasm, internal cancers	–	–

*BCC, Basal Cell Carcinoma; SCC, Spino Cell Carcinoma*.

##### Patient XP134

Patient XP134 was a 15-year old girl born from a consanguineous marriage (Figure 1B), with parents originating from the central region of Tunisia. The patient did not show any neurological abnormalities. As she was not protected from UV radiations, she presented an important number of actinic keratosis on sun-exposed areas and numerous squamous cell carcinomas and basal cell carcinomas on the face especially around the nose. She presented few hyperpigmented and hypopigmented patterns macules on her skin (Table 1). We tried to reach XP134's sister who was described as having similar clinical manifestations as pigmentation problems on sun exposed area, but she was out of reach. The proband's parents as well.

#### Genetic Investigations

##### Sanger Sequencing for Recurrent Mutations

Genetic pre-screening for recurrent and founder mutations observed in Tunisian XP patients was conducted in both patients XP21 and XP134 using Sanger Sequencing. Firstly, the patients were screened for already known mutations that are recurrent in Tunisian population as: p.Arg228^*^ in *XPA* gene, p.Val548AlafsX25 in *XPC* gene and the deletion g.36847_40771del3925 in *POLH* gene. Secondly, we screened the mutations associated with other mild forms of XP with neurological manifestations as: p.Arg683Gln in the *ERCC2* gene (XP-D phenotype), P.Leu778Pro in *ERCC5* gene (XP-G phenotype) and p.Lys381Argfs^*^2 mutation in *DDB2* gene (XP-E phenotype). None of these mutations was found.

Therefore, samples underwent targeted gene sequencing to cover other genes of the DNA repair pathways.

##### Targeted Gene Sequencing Results

**Figure 2:**
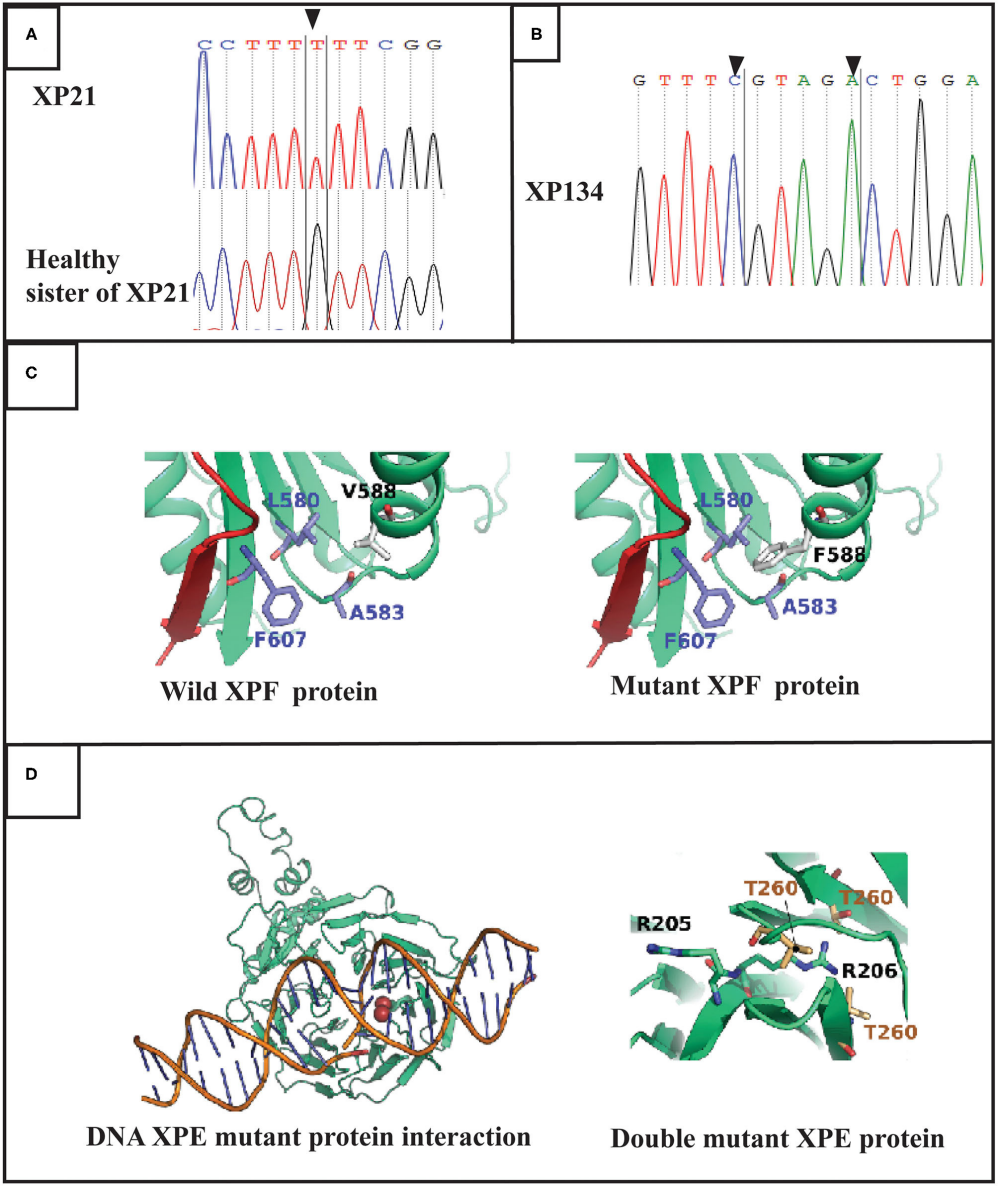
Molecular findings: **(A)** Electropherogram showing the variation in *ERCC4* gene in exon 8 (c.1762 G>T p.V588F) at a homozygous state in the patient XP21. **(B)** Electropherogram showing double variation in *DDB2* gene (c.613 T>C p.C205R and c.618 C>A p.S206R) in exon 5 in XP134 patient. **(C)** Structures of the XPF protein and F588 XPF mutant suggesting a mild distortion in helix domain. **(D)** DNA DDB2 protein interaction for muted DDB2 protein suggestion that R206 affect this process.

Through targeted gene sequencing of 87 genes involved in NER pathway, we identified in patient XP21 a novel homozygous missense variation in *ERCC4*, within exon 8, (NM_005236.2 c.1762G>T p.Val588Phe) (Figure 2A). Bioinformatic prediction tools (SIFT, POLYPHEN, mutation taster…) indicated that this mutation was pathogenic (Table 2). Moreover, through the prediction algorithm of human splicing finder tools, this variation was suggested to create an exonic splicing silencer (ESS) probably altering the splicing process.

**Table 2:** Prediction scores of the new detected variants in DDB2 and ERCC4 genes.

**Gene**	**Variant**	**Prediction tools**	**Score**	**Significance**
DDB2	c.613T>C; p.C205R	Mutation taster	0.99	Disease causing
		Polyphen	1	Probably damaging
		SIFT	2.77	damaging
		M-CAP	0.066	Possibly damaging
		Provean	−6.13	deleterious
		MUpro	−1.40	Decrease stability
DDB2	c.618C>A; p.S206R	Mutation taster	0.99	Disease causing
		Polyphen	1	Probably damaging
		SIFT	2.78	damaging
		M-CAP	0.05	Possibly damaging
		Provean	−2.9	deleterious
		MUpro	−1.05	Decrease stability
ERCC4	c.1762G>T; p.V588P	Mutation taster	0.99	Disease causing
		Polyphen	1	Probably damaging
		SIFT	2.8	damaging
		M-CAP	0.177	Possibly damaging
		Provean	−5.69	deleterious
		MUpro	−1.64	Decrease stability

Regarding the patient XP134, we found that he carried a double homozygous missense variation in *DDB2* gene in exon 5 (NM_000107.2: c.613T>C p.Cys205Arg) and c.618C>A p.Ser206Arg (rs759622121) (Figure 2B). Both variants were not described in any population database (Exac, GnomAD, 1000 Genomes.) and both are predicted to induce pathogenic effects using several prediction tools (SIFT, POLYPHEN, mutation taster…) (Table 2). Concerning the ACMG classification (Varsome), both variants were classed as of uncertain significance (class 3). Furthermore, this particular genetic profile was not previously described in any XP patient. Molecular diagnosis was confirmed by Sanger sequencing for each variation.

#### Molecular Modeling of the Mutant Protein Structures

##### Modeling XP-F Mutation

The search for a template using BLAST-based approaches was not able to identify an appropriate template. Therefore, we used a fold recognition based approach for such an end. First, we predicted the repartition of the domains of XPF along the sequence. We found that the segment 374–623 matches a hit with a significant E-value of 7.60e-98 corresponding to a helicase insert domain superfamily in accordance with the functional properties of XPF. We then proceeded to model this domain using a threading approach. pGenTHREADER was able to identify several hits with high confidence values. We selected the PDB entry, 1WP9, which corresponds to the crystal structure of Pyrococcus furiosus Hef helicase domain (PubMed: 15642269) as a template to construct the model. Our initial attempt to predict the structure led to a bad quality model consisting of severe distortions of the rigid secondary structure elements. In fact, the RMSD (Root Mean Square Distance) between the template and the model is of 6 Angstroms. When we inspected the model, we found that the problem is caused by the long exposed loops spanning between the alpha-helices of the protein. We, therefore, proceed by trimming the sequences of these loops (Supplemental Material) to keep at least 4 amino acids from the C and N termini ends. Such an approach might be capable of preserving the original properties of the exposed surface of the target protein. The Ramachandran plot confirmed a decent quality of the model with 90% of the dihedral angles are in the favored domain and 7% in the allowed region. Even with such a model, we could not be able to explain the effect of the mutation. Therefore, we inspected the possibility that the predicted structure of the helicase insert domain could establish interdomain contacts in the XPF protein. We proceeded first by predicting the residue-residue contact map using RaprorX (Wang et al., 2017). Indeed, the tool predicted a putative interaction of the helicase insert domain with residues 210-216 (EVVEIHV). According to the protein secondary structure prediction, this segment corresponds to a beta-sheet strand (Drozdetskiy et al., 2015). This region is also flanked by two alpha-helices with 6 and 24 residues. The equivalent aligned segment of the template structure corresponds to two helices of 5 and 24 amino acids flanking a beta stand segment interacting with the helicase insert domain.

Therefore, the structure of XPF was modeled in the presence of the 210-216 segment.

The position of V588 (R138 in the structure) corresponds to an alpha-helix (585-LTFVRQ LEIYR-596) rich in charged and polar amino acids. Residues of this helix are exposed to the solvent with only F589 and L591 making part of the hydrophilic core of the protein. We noticed that in the V588 in the wild type protein forms a hydrophobic cluster of residues with F607, A583, and L580. In the case of V588F mutation causes steric clashes with L580 residue due to its large bulky side chain compared to a valine amino acid in the wild type form (Figure 2C).

##### Modeling XP-E Mutation

The two mutations, C205R and S206R are located on a beta sheet that forms one of the blades of the WD40 beta-propeller domain. The mutations are close to the protein-DNA interface which are known to rely on electrostatic forces to stabilize the complex. In order to verify if the two mutations can disrupt the electrostatic properties of the protein-DNA interface, we calculated the pKa for the ionizable side chains at positions 205 and 206 for both the wild type and the mutant forms at physiological pH of 7.4 using PROPKA 3.0 (10.1021/ct100578z). The pKa value of C205 side chain is predicted to be at 8.59. Therefore, this residue is probably deprotonated in the wild type form. Both residues R205 and R206 in the mutant form are positively charged with predicted pKa values of 10.41 and 8.63. To our surprise, the electrostatic potential at the protein DNA interface did not drastically change between the wild type and the mutant forms (Supplementary Data). The R205 ionizable group remains exposed to the central cavity of the WD40 beta-propeller domain. The same configuration is also demonstrated by the side chain of C205 residue in the wild type form. However, for R206, the side chain is bulky compared to S206 amino acid in the wild type form. Its relatively long side chain is unable to interact with the deepest most residues forming the interface of two bladders (Residues V225, V245 and T260) which is not the case for S206 in the wild type form (Figure 2D).

## Materials and Methods

### Patients

This study was conducted according to the principles of the declaration of Helsinki and has obtained the ethical approval (IPT/LR05/ProjectPCI/22/2012/v2) from the institutional review board of Institut Pasteur de Tunis.

The two patients (XP21 and XP134) referred to dermatology department of Charles Nicolle hospital with clinical features suggestive of XP. Written informed consent was obtained for genetic analysis from patients or their parents.

### DNA Extraction, Library Construction, Sequencing, and Data Analysis

Genomic DNA was extracted from peripheral blood. Amplicon libraries were prepared from 1,000 ng of genomic DNA from patients. Custom design of DNA repair disorders' panel was performed using SureDesign (Agilent Technologies Inc.), with probes that cover the exons in 87 genes and 15 bp of the surrounding intronic sequences. Library preparation for NGS was done using the Agilent's HaloPlex^HS^ workflow as a target enrichment method. Massively parallel sequencing was performed on an Ion Torrent PGM (Thermo Fisher Scientific).

The data from the sequencing runs were analyzed using the Torrent Suite v4.0.2 analysis pipeline, and aligned using TMAPv.3. The output variant call format (VCF) file was then annotated using the in-house VarAft software version 2.5, which is available online (http://varaft.eu/index.php) (Desvignes et al., 2018). Sequence variants were compared with data in Exome Variant Server (http://evs.gs.washington.edu/EVS/), 1000 Genomes Project (http://www.1000genomes.org/), or GnomAD (https://gnomad.broadinstitute.org/), including Exome Aggregation Consortium database (ExAC), Cambridge, MA (http://exac.broadinstitute.org). A number of online tools were used to predict the functional impact and pathogenicity of the variants such as MutationTaster (http://www.mutationtaster.org/), PolyPhen (http://genetics.bwh.harvard.edu/pph2/), SIFT (http://sift.bii.a-star.edu.sg/), M-CAP (http://bejerano.stanford.edu/mcap/), Provean (http://provean.jcvi.org/index.php) and MU Pro for protein stability prediction (http://mupro.proteomics.ics.uci.edu/).

### Sanger Sequencing

The detected variants with a high prediction score of pathogenicity underwent targeted Sanger sequencing for validation using the following primers: **ERCC4F**: 5′GTAAGATGTCTTCCCTTCGG 3′; **ERCC4R:** 5′CATAAGCAGCATCGTAACGG 3′ and **DDB2F**: 5′ATGGAGCAGTCTGAATGTTC 3′; **DDB2R**: 5′CCACTCCTCTAGACAGG3′) which cover both variants in DDB2 gene. Direct sequencing was performed using Big Dye terminator technology (ABI 3130), and sequences were analyzed using Bioedit packages. Genomic and protein sequence variants are described following the Human Genome Variations Society Guidelines available at https://varnomen.hgvs.org/. *ERCC4* and *DDB2* variants are, respectively, described relative to the transcript reference sequences NM_005236.2 and NM_ NM_000107.2. ACMG classification of the variants (Richards et al., 2015) was obtained using Varsome at https://varsome.com.

### Protein Modeling

The primary structure of XPF endonuclease was deposited in UiprotKB database under the accession Q92889. The sequence consists of 916 amino acids of which only the structure corresponding to segment 834-916 was solved experimentally. To solve the structure of the region containing the mutation we first searched for conserved domains of the entire sequence. The search for the appropriate template to construct the 3D structure of XPF was performed using the pGenTHREADER program. The sequences of the best hits were then processed and aligned to the target sequence of XPF using 3D-expresso. The resulting alignment was then used to guide the model building process by MODELER (version 9.15). Twenty conformers were generated by the comparative modeling software of which we selected the best model according to the DOPE and the GA341 scores. Finally, the stereochemical quality of the model was assessed by constructing the Ramachandran plot to dress the repartition of the phi/psi angles in the favored, allowed, and non-tolerated dihedral domains.

The XP-E structure was retrieved from the co-crystal complex of PDB code 4E54 (PMID 22822215).

## Discussion

XP is a rare autosomal recessive disorder, characterized by ubiquitous defects in the DNA repair system. This disorder is especially characterized by sunburn after brief sun exposure and pigmented macules on sun-exposed skin (Krieger and Berneburg, 2012). Targeted gene panels help to focus on a set of relevant candidate genes within the overlapping phenotypes with similar clinical manifestations, to identify the exact genetic etiology while providing cost and time advantages when compared to classical Sanger sequencing.

About 40% of XP patients do not show sunburn reactions (Bradford et al., 2011). As for XP-E, increased number of freckles in sun-exposed areas seems to be the unique symptom for clinical diagnosis of the disease. These maculae are usually ignored and only the development of primary malignant lesions seems to be the reason leading to clinical consultation. This often results in a late diagnosis of patients with mild XP forms.

We report in the region, the XP-F form which is among the rarest complementation groups of XP. To date, 29 XP-F patients were reported worldwide (Ferri et al., 2020). Most of them are Japanese (Tofuku et al., 2015). XP-F (Omim: #278760) is caused by mutations in *ERCC4* gene. Furthermore, several *ERCC4* mutations were associated to a combined XP/CS phenotype, to XFE progeroid syndrome, Cockayne syndrome, Cerebellar ataxia-dominant phenotype or to Fanconi Anemia disease complementation group Q (Kashiyama et al., 2013; Doi et al., 2018). Although the clinical features of patient XP21 indicated a phenotype of Xeroderma pigmentosum, a moderate form of Fanconi anemia type Q was suspected. This hypothesis was quickly dismissed as patient XP21, and despite her short stature, did not develop internal cancers or neurological disorders. Fertility was not explored as the patient was not married.

The protein XPF consists of 916 amino acids. It forms a heterodimeric complex with ERCC1 and plays the role of structure-specific endonuclease 5' incision during the activation of NER repair system upon UV induced DNA damage (Sijbers et al., 1996; Richards et al., 2015). Twenty-seven variations have been reported thus far in the *ERCC4* gene (Zhou et al., 2017). While most mutations in this gene are located in exon 8, suggesting that it may represent a hotspot mutation site, the variants in this area can lead to different disorders. Indeed, patient XP21 harbored a novel G to T transversion at position c.1762. In the same exon, other variants as p.R589W give rise to combined XP/CS/FA, Fanconi anemia or mild XP forms. In our study, given the late onset of cutaneous cancer and the lack of associated neurological disorders, we suggest that patient XP21 was affected with a mild XP-F form.

The *ERCC4* endonuclease consists of an N-terminal DNA helicases domain (1-457), a domain containing the nuclease active site (656-813) and a C-terminal containing 2 tandem helix-hairpin-helix (HhH) domain (Manandhar et al., 2015). The previously reported *ERCC4* mutations are widely distributed over the entire length of the protein (Bogliolo et al., 2013; Kashiyama et al., 2013; Zhou et al., 2017; Mori et al., 2018). The novel reported variation in this study is located outside the nuclease active site.

The molecular modeling study suggests that the V588F mutation effect is probably caused by the steric clashes between F588 and L580. The latter amino acid belongs to the beta sheet which holds many of the core residues of the helicase insert domain. Of most importance, the 210-216 segment also belongs to this beta-sheet forming the outermost strand. The steric clash between F588 and L580 could cause a local distortion of the beta sheet. While this might not have a significant effect on the interaction of the helicase insert domain with the DNA, the fact that the 210-216 segment is implicated in the interaction network of the central beta sheet, suggests the cooperative functionality established between modeled helicase insert domain and other segments of the protein might be affected by the mutation.

Xeroderma pigmentosum type E (Omim: # 278740) caused by mutations in the *DDB2* gene. The DDB2 subunit is a component of a complex involved in ubiquitin-mediated proteolysis. Consisting of 427 residues, it plays a major role in DNA-damage recognition (Stoyanova et al., 2009). To date, more than 24 XP-E patients have been reported worldwide (Nichols et al., 1996; Itoh et al., 2000; Vahteristo et al., 2007; Oh et al., 2011; Karagün et al., 2020; Yang et al., 2020) and 17 variations were reported in *DDB2* gene (Yang et al., 2020). None of them is from North African countries except 3 Tunisian patients reported previously by our lab (Ben Rekaya et al., 2017). In this study, we report another novel variation in the Tunisian population emphasizing the complex genetic background of North African population.

It is a double homozygous patient harboring two novel mutations in the same exon of *DDB2* gene, responsible for XP-E, which was never described in XP patients. The emergence of rare autosomal genetic variants is frequent in people with common ethnic origin caused by consanguinity and endogamy. However, the appearance of double variations is an extremely rare event. These double mutations could explain the severe clinical phenotype in XP134 patient.

The DDB2 protein is composed from an N-terminal helix-loop helix segment (101 to 136) followed by a 7-bladed WD40 b propeller domain (residues 137 to 454) (Scrima et al., 2008). It is an ubiquitin ligase component of a multimeric complex involved in the degradation of DNA damage-response proteins (Liu et al., 2009). The two C205R and S206R variants in XP134 are located on a beta sheet that forms one of the blades of the beta-propeller WD40 domain of the DDB2 protein. Mutations close to the protein-DNA interface are known to delay electrostatic forces to stabilize the complex (Scrima et al., 2008), which could explain the severity of the XP134 phenotype compared to previously described XP-E patients (Ben Rekaya et al., 2017).

Our protein modeling study also suggests that the effect of the C205R mutation is not probably responsible for the clinical phenotype since the mutation preserves both, the number of charges carried by the corresponding residue and the electrostatic potential at the protein-DNA interface. Instead, the S206R mutation might have a significant effect on the protein structure. This could be explained by the fact that the mutation causes the R205 positively charged side chain to be buried inside the hydrophobic core of the protein. Such configuration is highly unstable and could cause the local distortion of the protein at the mutation level, but also could have a more severe consequence on the interaction with the damaged DNA.

## Conclusion

In this report, we further characterize new XP complementation groups emerging in the Tunisian population and in the region. While the mutational findings have improved the understanding of XP phenotype, it is necessary to continue the molecular investigations for other underdiagnosed patients for whom the discovery of genetic etiology will improve the clinical follow up.

## Data Availability Statement

The original contributions presented in the study are included in the article/Supplementary Material, further inquiries can be directed to the corresponding author/s.

## Ethics Statement

The studies involving human participants were reviewed and approved by Institut Pasteur Tunisia ethical committe. Written informed consent to participate in this study was provided by the participants' legal guardian/next of kin. Written informed consent was obtained from the minor(s)' legal guardian/next of kin for the publication of any potentially identifiable images or data included in this article.

## Author Contributions

IN did experiments. AC did the analysis, interpretations of data, and drafted the manuscript. HO did *in silico* protein modeling. SE and AL did experiments, analysis, and interpretation of targeted gene sequencing data. MJ and MZ clinical investigation of patients and family members. MR and OM helped in patients pre-screening for known mutations. VD, AD, NL, and SA study concept and design. AD, SA, and HY-Y critical revision of the manuscript. HY-Y supervision of the study. All authors contributed to the article and approved the submitted version.

## Conflict of Interest

The authors declare that the research was conducted in the absence of any commercial or financial relationships that could be construed as a potential conflict of interest.
