# Supplementary material for: Case Report: Identification of Novel Variants in ERCC4 and DDB2 Genes in Two Tunisian Patients With Atypical Xeroderma Pigmentosum Phenotype
Source: Front Genet. 2021 May 31;12:650639. doi: 10.3389/fgene.2021.650639 (PMC8203331; doi:10.3389/fgene.2021.650639)
Supplement: Supplementary file 1 [file Data_Sheet_1.docx]

**Supplementary material 1:**

This sequence was used to model the 3D structure of helicase insert domain from the sequence of human XPF protein.

>trimmed loops

VLESNPKWEALTEVLKEIEAENKESEAFLLRLYRKTFEKDSKAEEVWMKKKKKRKLTLTQMVGKPEELEEEGDVEEGYRREISSSPESCPEEIKHEEFDVNLSSDAAFGILKEPLTIIHLHEVEPRYVVLYDAELTFVRQLEIYRASRPGKPLRVYFLIYGGSTEEQRYLTAL

**Supplementary material 2:**


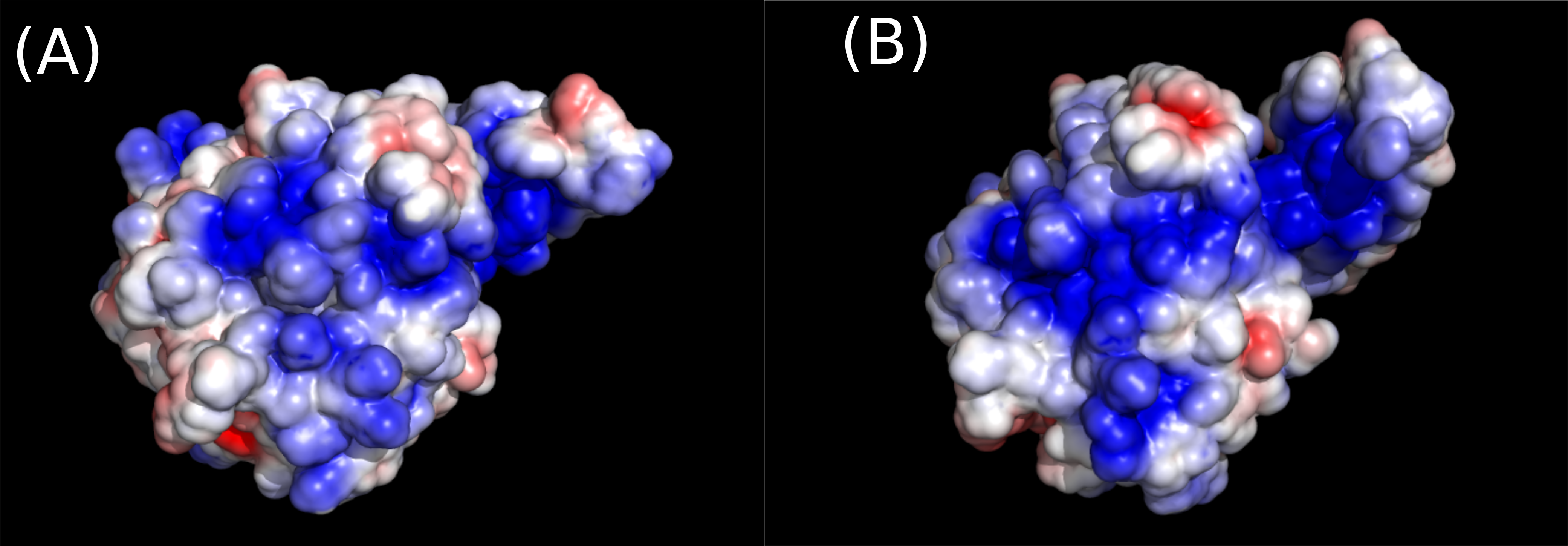


Calculation of the electrostatic potential. The intensity of the potential vary from -2 (red) kcal/(mol·e) to +2 kcal/(mol·e)(blue). We show the surface involved in the DNA interaction for the wild type form (A) and the mutant form of DDB2 protein(B)
